# Urinary free cortisol and childhood maltreatments in eating disorder patients: New evidence for an ecophenotype subgroup

**DOI:** 10.1002/erv.2896

**Published:** 2022-03-10

**Authors:** Paolo Meneguzzo, Cecilia Mancini, Samira Terlizzi, Chiara Sales, Maria Federica Francesconi, Patrizia Todisco

**Affiliations:** ^1^ Eating Disorders Unit Casa di Cura “Villa Margherita”, Arcugnano (VI) Vicenza Italy; ^2^ Department of Neuroscience University of Padova via Giustiniani 2 Padova Italy; ^3^ Padova Neuroscience Center University of Padova Padova Italy; ^4^ Experimental Medicine Department Food Science and Human Nutrition Research Unit Sapienza University of Rome Rome Italy

**Keywords:** 24‐h urinary free cortisol, child maltreatment, cortisol, eating disorder

## Abstract

**Introduction:**

Increasing neurobiological evidence has suggested the presence of a specific ecophenotype in people with eating disorders (EDs) linked to early maltreatment. Urinary‐free cortisol could strengthen the data and show specific relationships between maltreated subtypes and the hormonal profiles of patients with EDs. This study aims to evaluate the presence of different urinary cortisol in drug‐free patients in the acute phase of the disorder and its relationship with childhood maltreatment.

**Methods:**

A sample of 78 female patients with ED is included in the study. Childhood maltreatment history and 24‐h urinary free cortisol (24‐h UFC) are evaluated at a specialised ED ward admission.

**Results:**

Patients with a maltreatment history show more blunted 24‐h UFC levels than peers without childhood maltreatment (*p* = 0.001). Regression analysis showed that child abuse is a predictor of the reduction of 24‐h UFC (*p* < 0.001), with physical abuse (*p* = 0.011) and sexual abuse (*p* = 0.050) that could have a more specific impact than other maltreatment subtypes.

**Discussion:**

Childhood maltreatment should be evaluated in ED patients due to its biological impact on the hormonal stress axis, which could impair the ability of patients to respond to standardized ED treatment.

## Highlights

- Eating disorder patients with history of maltreatment showed a reduced 24‐h urinary free cortisol.
- New data support the evidence about the presence of an ecophenotype ED subgroup.
- Reduced 24‐h urinary free cortisol is significantly correlated with child abuses.

**Abbreviations**
BMI: body mass index
BTQ: brief trauma questionnaire
CTQ: Childhood Trauma Questionnaire
ED: eating disorder
EDE‐Q: eating disorder examination questionnaire
HPA: hypothalamus‐pituitary‐adrenal
UCRE: urinary creatinine
UFC: urinary free cortisol
UVOL: urine volume

## 1 INTRODUCTION

Traumatic events and posttraumatic symptomatology have shown a high prevalence in individuals with an eating disorder (ED) (Brewerton et al., 2020), with a specific influence of early maltreatment in the development of more severe psychopathology (Meneguzzo et al., 2021; Molendijk, Hoek, Brewerton, & Elzinga, 2017). Life stressors are thought to play a role in the onset of EDs (Favaro, 2013), and these stressors may occur from gestation through the early developmental moments (Favaro, Tenconi, & Santonastaso, 2006). Childhood exposure to maltreatment has been widely related to the development of an ED, with evidence of stable alterations of the body stress system (Caslini et al., 2016; Chami et al., 2019; Monteleone et al., 2021) that remain stable even after specific treatment protocol showing differences between childhood and adulthood maltreatment (Lelli et al., 2019; Meneguzzo et al., 2021). A growing body of literature has proposed the introduction of the construct of a maltreated ecophenotype in psychiatric research due to the structural and functional alterations in the brain found as a consequence of early stressful environmental experiences (Teicher & Samson, 2013), even in the ED field (Monteleone et al., 2021). However, few studies have evaluated the biological effects of early maltreatment in patients with an ED, despite the presence of robust transdiagnostic evidence of shared neurobiology (Frank, Shott, & DeGuzman, 2019).

The neurobiological hypothesis proposed by previous literature is that early traumatic experiences may increase the risk of EDs due to modification of the body's stress response system (Het et al., 2015). Indeed, both the sympathetic nervous system—with the reduction of the secretion of catecholamines—and the hypothalamus‐pituitary‐adrenal (HPA) axis—with the reduction of the cortisol secretion—have shown an impaired function in maltreated ED patients compared to non‐maltreated ones (Cascino et al., 2020; Monteleone et al., 2020). Focussing on the HPA axis, a reduction of the salivary cortisol awakening response and a reduced of the salivary cortisol response to a specific social stressor have been reported in ED patients with a history of trauma, with differences among patients with and without a history of early maltreatment (Monteleone et al., 2018). According to the posttraumatic literature, urinary cortisol could be a valid biomarker providing an integrated measure that might be more trustworthy in showing hormonal changes than random salivary samples (Pan et al., 2020). Measuring 24‐h urinary free cortisol (24‐h UFC) could be a valid methodology to assess daily pulses of the HPA axis and the basal production of cortisol (El‐Farhan et al., 2017) and is moderately stable both in the long and short terms. In the posttraumatic literature, 24‐h UFC has consistently demonstrated differences between trauma‐exposed people and not, with more robust differences found in subjects who have experienced physical or sexual abuse (Li & Seng, 2018; Schumacher et al., 2019). In the ED literature, robust data have indicated significant differences in urinary cortisol in women with ED compared to healthy peers, with increased cortisol levels in severe and acute phases (e.g., during fasting and pathological weight loss or during a period of binging and vomiting). However, preliminary data have shown reduced cortisol excretion over the day in patients with a traumatic history (Koo‐Loeb et al., 2000; Lo Sauro et al., 2008), with inconclusive results. Indeed, these data showed specific dysregulation of the HPA axis, but results are still preliminary, and new evidence is needed to evaluate the neurobiological effects of environmental events in ED patients (Chami et al., 2019; Lo Sauro et al., 2008).

Thus, this study aims to evaluate basal differences in the 24‐h UFC between ED patients with and without maltreatment histories. The hypothesis is that patients with previous childhood maltreatment will present a reduced daily excretion of cortisol compared to patients without a history of maltreatment. This could add data to the hypothesis of the presence of a specific ecophenotype in ED patients with a history of childhood trauma, calling for a specific evaluation of clinical presentation and treatment efficacy. Our secondary objective is to evaluate relationships between UFC and specific forms of maltreatment.

## 2 METHODS

### 2.1 Participants

Consecutive patients admitted to the inpatient Eating Disorder Unit of the Casa di Cura Villa Margherita (Arcugnano, VI‐Italy) from July 2019 to May 2021 were enroled in the present study.

According to the existing neurobiological literature about ED patients (Chami et al., 2019), the inclusion criteria were (1) a diagnosis of anorexia nervosa or bulimia nervosa; (2) age between 16 and 50 years old; (3) medical stability. All the participants were evaluated by a trained psychiatrist before admission to the facility, and their diagnoses were formulated according to the criteria of the Diagnostic and Statistical Manual of Mental Disorders 5 (American Psychiatric Association, 2013). Patients accessed the inpatient facilities for psychiatric acuity after the failure of at least one outpatient treatment. The exclusion criteria were (1) a prior or current traumatic brain injury, (2) lifetime history of any neurological or medical condition, (3) severe comorbid psychiatric illness (suicidality, alcohol/substance use, psychotic features), (4) medical comorbidities that require any drug treatment (e.g., hypothyroidism or hypertension), and (5) drug treatment with corticosteroids, oestrogen/progestin therapy or psychoactive medication in the previous 4 weeks.

A total sample of 78 participants with ED diagnoses agreed to participate: 49 patients with diagnoses of anorexia nervosa (AN: 29 with the restrictive subtype), with a mean age of 24.74 ± 9.74 years old [16–40] and a mean BMI of 16.15 ± 3.77; and 29 patients with a diagnosis of bulimia nervosa (BN), with a mean age of 23.65 ± 7.40 years old [16–38] and a mean BMI of 21.94 ± 4.16.

All participants gave written informed consent during their recruitment, or it was collected from their parents if the patients were under 18 years old. The study was in accordance with the ethical standard of the Declaration of Helsinki and was approved by the local ethics committee as part of the clinical evaluation of the patients.

### 2.2 Measures

Demographic and clinical measures (high and weight) were collected at the enrolment. The duration of illness was retrospectively defined as the time elapsed between admission to the ward and the onset of eating symptoms reported by the patient and her outpatient referrers.

Eating disorder psychopathology was evaluated using the eating disorder examination questionnaire (EDE‐Q), which contains 28 self‐report items about specific aspects of eating disorder psychopathology with 4 specific subscales—restraint, eating, shape, and weight concern—and a global score (Calugi et al., 2017).

General psychopathology was evaluated using the revised symptom checklist (SCL‐90R), which contains 90 self‐report items and is widely recognized as a valid tool to assess psychological distress levels (Derogatis et al., 1974). In addition to the original subscales from the original 90‐item questionnaire, the literature has demonstrated that a posttraumatic stress disorder (PTSD) subscale could be used (Carlozzi & Long, 2008).

The brief trauma questionnaire (BTQ) was used to screen for traumatic life events (Schnurr et al., 1999). This is a 10‐item self‐report questionnaire used to evaluate exposure to 10 traumatic events. Possible scores range from 0 (no trauma) to 10 (traumatic experiences in each investigated category).

Childhood trauma was assessed with the short form of the Childhood Trauma Questionnaire (CTQ), a 28‐item questionnaire with five subscales that evaluate five specific forms of childhood trauma: sexual abuse, physical abuse, physical neglect, emotional abuse, and emotional neglect (Bernstein et al., 2003). Subjects scoring higher than the threshold of at least one subscale were classified as participants with maltreatment history (M+). Those who scored below the thresholds for all five subscales were classified as participants with no maltreatment history (M−). The previously validated cut‐offs of each subscale were: sexual abuse ≥8, physical abuse ≥8, physical neglect ≥8, emotional abuse ≥10, and emotional neglect ≥15 (Walker et al., 1999).

Urinary‐free cortisol (μg/24 h) concentrations were determined using a coated‐tube radio‐immunoassay (Siemens Healthcare) (intraassay coefficient of variation, 5.1%; interassay coefficient of variation, 8.4%). The assay was performed according to the manufacture's instruction using 0.5 ml urine and included a preceding extraction step with dichloromethane. The 24‐h urinary creatinine (24‐h UCRE, mg/24h) was evaluated using a colourimetric kit based upon the Jaffe reaction (sensitivity = 0.042 mg/dl).

### 2.3 Procedure

All participants were evaluated with self‐report questionnaires during the first week of their admission to the ward. There were asked to collect 24‐h urine samples 2 days after admission, from 8 to 8 AM of the day after. Menstruating women were asked to collect their 24‐h urine samples on the seventh day following the start of menses to ensure that the plasma oestrogen milieu was as close as possible to that of non‐menstruating women. Urinary samples were stored in dark bottles during the collection and preserved in a refrigerator before analysis.

### 2.4 Statistics

Differences between M+ and M− subgroups were evaluated with *t*‐tests for independent samples, and 24‐h UFC analysis was confirmed with an ANCOVA analysis with 24‐h UCRE and 24‐h urine volume (24‐h UVOL, ml/24 h) as covariates due to the relationship between urine cortisol and creatinine reported in the literature. A chi‐square analysis was used to evaluate differences in the distribution of lifetime traumatic events and amenorrhoea between subgroups. Cohen's *d* and *η* ^*2*^ were used for the evaluation of effect sizes. Hierarchical regression analyses were performed to evaluate the presence of a relationship between specific traumas and 24‐h UFC (model 1). In the second model, we included nutritional status (BMI). The third model included the specific eating psychopathological score (EDE‐Q). We used this approach to evaluate the covariance role of physical and psychological aspects that could effects daily cortisol secretion. Moreover, for all the participants, we evaluated early maltreatment as a spectrum, rather than a dichotomous condition. Linear regression analyses were applied to evaluate the relationship between 24‐h UFC and specific CTQ subscales. To control for multiple analyses, we applied the Bonferroni correction, and we considered significant *p*‐values to be ≤0.017. All the statistical analyses were performed with SPSS version 25.0.

## 3 RESULTS

No significant differences were found in the distribution of differential diagnoses among patients with and without histories of child maltreatment. The M+ subgroup included 24 of the 49 patients with diagnoses of AN and 16 of the 29 patients with diagnoses of BN. Looking at the lifetime presence of traumatic events according to the BTQ scores, no differences were found between the M+ and M− groups distribution: 5 patients in the M− group and 11 patients in the M+ group reported lifetime traumatic events [*χ* ^*2*^ (1) = 2.458, *p* = 0.117].

No significant differences emerged between M+ and M− regarding age (M+: 23.50 ± 9.40 years, M−: 21.76 ± 5.64 years, *t* (49) = −0.916, *p* = 0.364), BMI (M+: 17.67 ± 6.38 kg/m^2^, M−: 18.71 ± 4.83 kg/m^2^, *t*(55) = 0.695, *p* = 0.490), amenorrhoea (M+: 60.0%, M−: 55.2%, *χ* ^*2*^ (1) = 0.280, *p* = 0.597), or duration of their disorders (M+: 3.20 ± 2.96 years, M−: 3.42 ± 2.76 years, *t*(76) = 0.341, *p* = 0.734). Psychological evaluation showed higher mean scores in M+ subgroup for all the variables considered, as can be seen in Table 1.

**TABLE 1:** Psychological evaluation of the participants divided by trauma history at the admission to the ward

		M− *n* = 38	M+ *n* = 40	*t*	*p*	*d*
EDE‐Q						
	Restraint	3.07 (2.05)	4.30 (1.59)	−2.820	**0.006**	0.671
	Eating concern	3.32 (1.56)	3.99 (1.13)	−2.090	0.040	0.494
	Shape concern	4.28 (1.49)	4.87 (1.02)	−1.933	0.058	0.462
	Weight concern	3.82 (1.60)	4.35 (1.42)	−1.495	0.139	0.350
	Global	3.57 (1.44)	4.38 (0.99)	−2.782	**0.007**	0.656
SCL‐90R						
	Depression	2.21 (0.69)	2.61 (0.54)	−2.786	**0.007**	0.646
	Anxiety	1.80 (0.83)	2.28 (0.75)	−2.715	**0.008**	0.607
	Psychoticism	1.49 (0.66)	1.35 (0.73)	0.879	0.382	0.201
	Obsessive‐compulsive	2.24 (0.82)	2.09 (0.75)	0.826	0.412	0.191
	Somatisation	1.65 (0.88)	1.79 (0.70)	−0.827	0.411	0.176
	Interpersonal sensitivity	2.24 (0.79)	2.14 (0.92)	0.491	0.625	0.117
	Hostility	1.32 (0.72)	1.16 (0.81)	0.881	0.381	0.209
	Phobic anxiety	1.39 (0.98)	1.07 (0.79)	1.581	0.118	0.360
	Paranoid ideation	1.72 (0.91)	1.63 (0.86)	0.486	0.628	0.102
	GSI	1.68 (0.58)	2.08 (0.50)	−3.204	**0.002**	0.739
	PTSD	2.39 (0.83)	2.62 (0.74)	−1.334	0.186	0.292
CTQ						
	Emotional abuse	5.47 (1.06)	12.45 (4.65)	−9.237	**<0.001**	2.070
	Physical abuse	5.08 (0.27)	7.38 (3.64)	−3.983	**<0.001**	0.891
	Sexual abuse	5.03 (0.16)	7.93 (4.63)	−3.960	**<0.001**	0.882
	Emotional neglect	6.95 (1.79)	15.68 (5.20)	−10.011	**<0.001**	2.245
	Physical neglect	5.16 (0.44)	7.70 (2.64)	−5.998	**<0.001**	1.342
	Total score	27.68 (2.82)	51.13 (13.38)	−10.833	**<0.001**	2.425

*Note*: M−: subgroup with no history of trauma; M+: subgroup with history of trauma. Significant differences were reported with bold characters.

Abbreviations: CTQ, Childhood Trauma Questionnaire; *d*, Cohen's *d*; EDE‐Q, eating disorder examination questionnaire; GSI, global severity index; PTSD, posM‐traumatic stress disorder.

**FIGURE 1:**
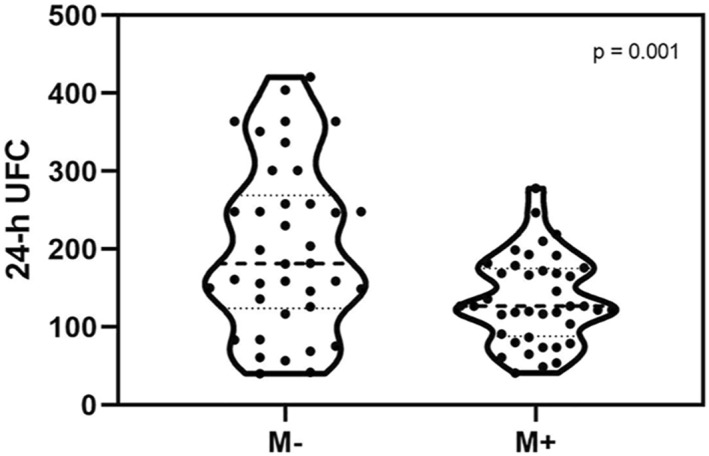
Graphical representation of the distribution of 24‐h UFC in the participants. The M− subgroup showed a higher excretion of urinal cortisol at the admission in the ward than M+, which presented a blunt cortisol urinal excretion

Looking at the urine analysis, we found no significant differences regarding 24‐h UCRE (M+: 0.927 ± 0.297, M−: 0.868 ± 0.575, *t* (76) = −0.572, *p* = 0.569) or 24‐h UVOL (M+: 1730.62 ± 724.80, M−: 1832.89 ± 1009.23, *t* (76) = 0.516, *p* = 0.607). Significant differences were found between M+ and M−looking at 24‐h UFC, with a reduced cortisol excretion in the M+ simple: M+ 135.03 ± 55.92, M−: 202.13 ± 108.02, *t* (55) = 3.419, *p* = 0.001, *d* = 0.780. This result was confirmed with an ANCOVA with 24‐h UCRE and 24‐h UVOL as covariate: *F* (1,74) = 11.544, *p* = 0.001, *η* ^*2*^ = 0.137. The result was also confirmed excluding participants with a history of traumatic events as screened with the BTQ: M+: 147.77 ± 56.90, M−: 209.56 ± 112.26, *t* (49) = 2.781, *p* = 0.008. See Figure 1 for a graphical representation of the 24‐h UFC distribution.

### 3.1 Regression analyses

**FIGURE 2:**
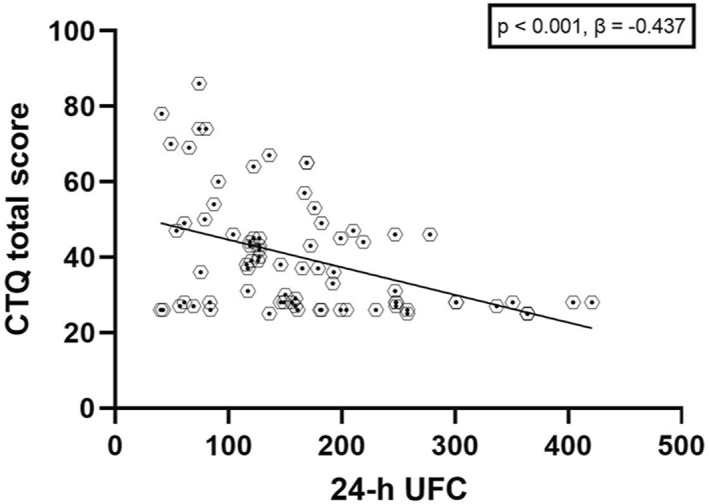
Graphical representation of the regression analysis with all the ED participants together. A clear relationship between the CTQ scores and the 24‐h UFC emerged, showing a negative effect of childhood maltreatments over the cortisol excretion in ED patients

Considering all the ED participants together, we found that the total score on the CTQ significantly predicted 24‐h UFC values: F (1,76) = 17.948, *p* < 0.001, *β* = −0.437, *R* ^2^ = 0.180. The inclusion in the regression model of BMI (*β* = −0.126, *p* = 0.322) and EDE‐Q total score (*β* = −0.077, *p* = 0.541) did not change the significance of the regression model, but no significant effects were found (Model 2: F (2,50) = 6.137, *p* = 0.004, Δ*R* ^2^ = 0.005; Model 3: F (3,49) = 4.190, *p* = 0.010, Δ*R* ^2^ = 0.007). See Figure 2 for a graphic representation of the model. A significant relationship was also found between the 24‐h UFC and the number of significant early maltreatment episodes, as scored with the CTQ cut‐offs: F (1, 76) = 14.130, *p* < 0.001, *β* = −0.396, *R* ^2^ = 0.146.

Linear regression analysis showed that CTQ subscales significantly predicted 24‐h UFC values (*R* ^2^ = 0.295, F (5,72) = 7.458, *p* < 0.001). Only physical abuse significantly explained 24‐h UFC (*β* = −0.325, *p* = 0.011). The results showed only a tendency for significant results with sexual abuse (*β* = −0.232, *p* = 0.050). No significant result was found for the other subscales (physical neglect: *β* = 0.181, *p* = 0.293; emotional abuse: *β* = −0.031, *p* = 0.841; emotional neglect: *β* = −329, *p* = 0.080).

Looking at the possible presence of a relationship between posttraumatic symptomatology and 24‐h UFC, no significant results were found in a linear regression with SCL90R‐PTSD scores as the independent variables: F (1,76) = 3.340, *p* = 0.073. Similarly, no significant relationship was found between 24‐h UFC and the other subscales of the SCL90‐R [F (11,62) = 1.213, *p* = 0.297].

## 4 DISCUSSION

In the present study, we used the 24‐h UFC to measure the HPA axis function in ED patients, searching for differences related to the presence or absence of childhood maltreatment. Our main finding was that patients with a history of maltreatment presented lower levels of 24‐h UFC than patients without childhood maltreatment, with no influence of age, BMI, or duration of the disorder. Moreover, our data showed a significant negative relationship between early maltreatment and 24‐h urinary cortisol, also considering all the individuals together. These findings are consistent with and add weight to previous literature showing an impaired awakening of the salivary cortisol response in ED patients with trauma (Monteleone et al., 2015, 2018), corroborating the hypothesis of a specific ecophenotype of ED patients with traumatic childhood histories.

Data from the literature highlighted the presence of impaired functioning of the HPA axis in ED patients during the acute phase of the disorder, even if mixed results were reported in different studies (Lo Sauro et al., 2008). Indeed, studies without an evaluation of the traumatic history of the participants have reported both the presence of an increased cortisol secretion if compared to healthy peers and a blunted one, with preliminary data that has proposed the role of early maltreatment as a possible explanation (Lo Sauro et al., 2008). Studies are limited, and our data corroborated this evidence, possibly explaining the mixed results presented in the literature.

Hypoactivity of the HPA axis in ED patients with a history of early maltreatment aligns with literature about early exposure to trauma and its effects on adults' responses to stressful events (Monteleone et al., 2020). This hypoactivity can be linked to stress systems exhausted by the long‐lasting effects of childhood maltreatment (Carpenter et al., 2009). In line with the bio‐psycho‐social and developmental models of EDs (Favaro, 2013; Frank, 2016), childhood trauma could impact biological traits such as increased sensitivity to salient stimuli or heightened anxiety, thus causing physiological stress responses to become inadequate. Moreover, childhood maltreatment could have lasting adverse effects on patients' general health because it could increase the presence of inflammatory cytokines already present in ED patients (Dalton et al., 2018) and might reduce the effect of psychotherapies (Castellini et al., 2018). Preliminary evidence of reducing 24‐h urinary cortisol in individuals with posttraumatic symptoms has been reported in the literature (Pan et al., 2020), with mixed results possibly due to small sample sizes and other confounders. However, our data highlighted the possibility that even without apparent posttraumatic symptomatology, individuals with a history of early maltreatment could display neurobiological features in the ED field.

Interestingly, the reduction of UFC was related to physical abuse in our sample, with a tendency also for experienced sexual abuse. Our findings lend additional information about the possible role of trauma subtypes in the neurobiology of ED patients, showing a possible specific effect of physical abuse on daily cortisol production, as suggested by previous preliminary literature data (Carpenter et al., 2009). Physical abuse represents a subtype of maltreatment that has been implicated in disturbing familiar dynamics (Klump et al., 2002; Schmidt et al., 1997), suggesting a possible vulnerability due to a troubled environment and impairment in attachment security and personal development. We did not find a clear relationship between UFC and sexual abuse, despite the high association reported in the literature (Madowitz et al., 2015). This could have been linked to the sample included or to the difficulty of individuals in reporting sexual abuse. Future studies should evaluate specific methodologies that could help detect sexual abuse.

From a clinical point of view, patients with traumatic experience have already been associated with more compromised psychopathological profiles (Meneguzzo et al., 2021) and have already shown the presence of impaired outcomes to standardized treatment (Castellini et al., 2018). For this reason, as already shown for other dysfunctional traits such as impulsivity in ED patients (Todisco et al., 2020), the presence of an ecophenotype for maltreated ED patients should push researchers and clinicians to evaluate personalised treatment approaches, as preliminary data have suggested (Chami et al., 2019). Indeed, looking from a longitudinal perspective, patients without a trauma history showed a significant reduction of cortisol excretion after specific treatment, but patients maltreated during their childhoods did not, corroborating the presence of neurobiological differences between patients with and without childhood maltreatment (Lelli et al., 2019). The presence of a neurobiological marker adds new strength to the argument that trauma‐informed treatments for ED patients could improve the outcomes of patients with severe trauma comorbidity (Brewerton, 2019; Rienecke et al., 2021). However, our data do not support the use of 24‐h urinary cortisol as a marker in clinical practice because maltreated and not‐maltreated subgroups fell into the same range of urinal cortisol without a clear distinction.

The strength of this study is its inclusion of an adequate sample of female patients not taking any medication and in a severely acute phase of their EDs. However, several limits should be considered. First, the participants collected the urinary samples after in‐depth instructions, but the procedure was not under strict observation in our clinical research unit. Second, both the BTQ and the CTQ were self‐report questionnaires and could have been affected by psychological aspects, including forgetting, dissociation, or reporting biases, limiting the results. Third, we did not use a specific questionnaire to evaluate posttraumatic stress disorder psychopathology, which might have affected the results. Future studies should include specific evaluations of posttraumatic stress disorder. Finally, the included sample represented female patients who had experienced the failure of at least one outpatient treatment, which could have influenced the severity of the psychopathology of the sample. Future studies should also include different genders, outpatients, and individuals with different ED diagnoses.

## 5 CONCLUSION

In conclusion, this study increases the evidence of a specific impact of childhood trauma in patients with EDs, presenting neurobiological data that support the idea of the presence of a specific ecophenotype. Blunt cortisol responses in acute phases of an ED could compromise treatment outcomes and the ability of patients to respond to standardized treatment protocols. However, more studies are needed, especially with longitudinal designs, that could also explain the relationship between biological features of patients with EDs and treatment outcomes. Our data highlights the possible relationship between low cortisol levels and childhood physical trauma in acute phases. Still, confirmatory studies are needed, even in patients with different diagnoses (i.e., binge eating disorder).

## CONFLICTS OF INTEREST

On behalf of all authors, the corresponding author states that there is no conflict of interest.

## AUTHOR CONTRIBUTIONS

Concept and design: Dr. Meneguzzo, Dr. Mancini and Dr. Todisco. Data collection: Dr. Mancini, Dr. Terlizzi, Dr. Sales, Dr. Francesconi, Dr. Meneguzzo. Analysis or interpretation of data: Dr. Meneguzzo and Dr. Todisco. Drafting of the manuscript: Dr. Meneguzzo and Dr. Todisco. Critical revision of the manuscript: all authors. Statistical analysis: Dr. Meneguzzo. Administrative, technical, or material support: Dr. Todisco. All authors approved the final version of the manuscript.

## CONSENT STATEMENT

Written informed consent was obtained from all participants or parents if underage.

## Data Availability

The data that support the findings of this study are available on request from the corresponding author. The data are not publicly available due to privacy or ethical restrictions.

## References

[erv2896-bib-0001] American Psychiatric Association . (2013). Diagnostic and statistical manual of mental disorders (DSM‐5). American Psychiatric Association. Washington, DC.

[erv2896-bib-0002] Bernstein, D. P. , Stein, J. A. , Newcomb, M. D. , Walker, E. , Pogge, D. , Ahluvalia, T. , Stokes, J. , Handelsman, L. , Medrano, M. , Desmond, D. , & Zule, W. (2003). Development and validation of a brief screening version of the Childhood Trauma Questionnaire. Child Abuse & Neglect, 27(2), 169–190. 10.1016/S0145-2134(02)00541-0 12615092

[erv2896-bib-0003] Brewerton, T. D. (2019). An overview of trauma‐informed care and practice for eating disorders. Journal of Aggression, Maltreatment & Trauma, 28(4), 445–462. 10.1080/10926771.2018.1532940

[erv2896-bib-0004] Brewerton, T. D. , Perlman, M. M. , Gavidia, I. , Suro, G. , Genet, J. , & Bunnell, D. W. (2020). The association of traumatic events and posttraumatic stress disorder with greater eating disorder and comorbid symptom severity in residential eating disorder treatment centers. International Journal of Eating Disorders, 53(12), 2061–2066. 10.1002/eat.23401 33159362

[erv2896-bib-0005] Calugi, S. , Milanese, C. , Sartirana, M. , El Ghoch, M. , Sartori, F. , Geccherle, E. , Coppini, A. , Franchini, C. , & Dalle Grave, R. (2017). The eating disorder examination questionnaire: Reliability and validity of the Italian version. Eating and Weight Disorders, 22(3), 509–514. 10.1007/s40519-016-0276-6 27039107

[erv2896-bib-0006] Carlozzi, N. E. , & Long, P. J. (2008). Reliability and validity of the SCL‐90‐R PTSD subscale. Journal of Interpersonal Violence, 23(9), 1162–1176. 10.4324/9781410602923-6 18292401

[erv2896-bib-0007] Carpenter, L. L. , Tyrka, A. R. , Ross, N. S. , Khoury, L. , Anderson, G. M. , & Price, L. H. (2009). Effect of childhood emotional abuse and age on cortisol responsivity in adulthood. Biological Psychiatry, 66(1), 69–75. 10.1016/j.biopsych.2009.02.030 19375070 PMC2696583

[erv2896-bib-0008] Cascino, G. , Monteleone, A. M. , Marciello, F. , Pellegrino, F. , Ruzzi, V. , & Monteleone, P. (2020). Alexithymia and cortisol awakening response in people with eating disorders. World Journal of Biological Psychiatry, 22(7), 1–551. 10.1080/15622975.2020.1844291 33135561

[erv2896-bib-0009] Caslini, M. , Bartoli, F. , Crocamo, C. , Dakanalis, A. , Clerici, M. , & Carrà, G. (2016). Disentangling the association between child abuse and eating disorders: A systematic review and meta‐analysis. Psychosomatic Medicine, 78(Issue 1), 79–90. 10.1097/PSY.0000000000000233 26461853

[erv2896-bib-0010] Castellini, G. , Lelli, L. , Cassioli, E. , Ciampi, E. , Zamponi, F. , Campone, B. , Monteleone, A. M. , & Ricca, V. (2018). Different outcomes, psychopathological features, and comorbidities in patients with eating disorders reporting childhood abuse: A 3‐year follow‐up study. European Eating Disorders Review, 26(3), 217–229. 10.1002/erv.2586 29542195

[erv2896-bib-0011] Chami, R. , Monteleone, A. M. , Treasure, J. , & Monteleone, P. (2019). Stress hormones and eating disorders. Molecular and Cellular Endocrinology, 497(July), 110349. 10.1016/j.mce.2018.12.009.2018 30557597

[erv2896-bib-0012] Dalton, B. , Bartholdy, S. , Robinson, L. , Solmi, M. , Ibrahim, M. A. A. , Breen, G. , Schmidt, U. , & Himmerich, H. (2018). A meta‐analysis of cytokine concentrations in eating disorders. Journal of Psychiatric Research, 103(March), 252–264. 10.1016/j.jpsychires.2018.06.002 29906710

[erv2896-bib-0013] Derogatis, L. R. , Lipman, R. S. , Rickels, K. , Uhlenhuth, E. H. , & Covi, L. (1974). The Hopkins symptom checklist (HSCL): A self‐report symptom inventory. Behavioral Science, 19(1), 1–15. 4808738 10.1002/bs.3830190102

[erv2896-bib-0014] El‐Farhan, N. , Rees, D. A. , & Evans, C. (2017). Measuring cortisol in serum, urine and saliva – are our assays good enough? Annals of Clinical Biochemistry, 54(3), 308–322. 10.1177/0004563216687335 28068807

[erv2896-bib-0015] Favaro, A. (2013). Brain development and neurocircuit modeling are the interface between genetic/environmental risk factors and eating disorders. A commentary on keel & forney and friederich et al. International Journal of Eating Disorders, 46(5), 443–446. 10.1002/eat.22131 23658088

[erv2896-bib-0016] Favaro, A. , Tenconi, E. , & Santonastaso, P. (2006). Perinatal factors and the risk of developing anorexia nervosa and bulimia nervosa. Archives of General Psychiatry, 63(1), 82–88. 10.1001/archpsyc.63.1.82 16389201

[erv2896-bib-0017] Frank, G. K. W. (2016). The perfect storm ‐ a bio‐psycho‐social risk model for developing and maintaining eating disorders. Frontiers in Behavioral Neuroscience, 10(MAR), 1–4. 10.3389/fnbeh.2016.00044 27014006 PMC4785136

[erv2896-bib-0018] Frank, G. K. W. , Shott, M. E. , & DeGuzman, M. C. (2019). The neurobiology of eating disorders. Child and Adolescent Psychiatric Clinics of North America, 28(4), 629–640. 10.1016/j.chc.2019.05.007 31443880 PMC6709695

[erv2896-bib-0019] Het, S. , Vocks, S. , Wolf, J. M. , Hammelstein, P. , Herpertz, S. , & Wolf, O. T. (2015). Blunted neuroendocrine stress reactivity in young women with eating disorders. Journal of Psychosomatic Research, 78(3), 260–267. 10.1016/j.jpsychores.2014.11.001 25499617

[erv2896-bib-0020] Klump, K. L. , Wonderlich, S. , Lehoux, P. , Lilenfeld, L. R. R. , & Bulik, C. (2002). Does environment matter? A review of nonshared environment and eating disorders. International Journal of Eating Disorders, 31(2), 118–135. 11920974 10.1002/eat.10024

[erv2896-bib-0021] Koo‐Loeb, J. H. , Costello, N. , Light, K. C. , & Girdler, S. S. (2000). Women with eating disorder tendencies display altered cardiovascular, neuroendocrine, and psychosocial profiles. Psychosomatic Medicine, 62(4), 539–548. 10.1097/00006842-200007000-00013 10949100

[erv2896-bib-0022] Lelli, L. , Castellini, G. , Cassioli, E. , Monteleone, A. M. , & Ricca, V. (2019). Cortisol levels before and after cognitive behavioural therapy in patients with eating disorders reporting childhood abuse: A follow‐up study. Psychiatry Research, 275(May), 269–275. 10.1016/j.psychres.2019.03.046.2018 30952070

[erv2896-bib-0023] Li, Y. , & Seng, J. S. (2018). Child maltreatment trauma, posttraumatic stress disorder, and cortisol levels in women: A literature review. Journal of the American Psychiatric Nurses Association, 24(1), 35–44. 10.1177/1078390317710313 28569082

[erv2896-bib-0024] Lo Sauro, C. , Ravaldi, C. , Cabras, P. L. , Faravelli, C. , & Ricca, V. (2008). Stress, hypothalamic‐pituitary‐adrenal axis and eating disorders. Neuropsychobiology, 57(3), 95–115. 10.1159/000138912 18552511

[erv2896-bib-0025] Madowitz, J. , Matheson, B. E. , & Liang, J. (2015). The relationship between eating disorders and sexual trauma. Eating and Weight Disorders, 20(3), 281–293. 10.1007/s40519-015-0195-y 25976911

[erv2896-bib-0026] Meneguzzo, P. , Cazzola, C. , Castegnaro, R. , Buscaglia, F. , Bucci, E. , Pillan, A. , Garolla, A. , Bonello, E. , & Todisco, P. (2021). Associations between trauma, early maladaptive schemas, personality traits, and clinical severity in eating disorder patients: A clinical presentation and mediation analysis. Frontiers in Psychology, 12(March). 10.3389/fpsyg.2021.661924 PMC8044897 33868136

[erv2896-bib-0027] Molendijk, M. L. , Hoek, H. W. , Brewerton, T. D. , & Elzinga, B. M. (2017). Childhood maltreatment and eating disorder pathology: A systematic review and dose‐response meta‐analysis. Psychological Medicine, 47(8), 1402–1416. 10.1017/S0033291716003561 28100288

[erv2896-bib-0028] Monteleone, A. M. , Cascino, G. , Ruzzi, V. , Pellegrino, F. , Patriciello, G. , Barone, E. , Carfagno, M. , Monteleone, P. , & Maj, M. (2021). Emotional traumatic experiences significantly contribute to identify a maltreated ecophenotype sub‐group in eating disorders: Experimental evidence. European Eating Disorders Review, 29(2), 269–280. 33378110 10.1002/erv.2818

[erv2896-bib-0029] Monteleone, A. M. , Marciello, F. , Cascino, G. , Cimino, M. , Ruzzi, V. , Pellegrino, F. , Del Giorno, C. , & Monteleone, P. (2020). Early traumatic experiences impair the functioning of both components of the endogenous stress response system in adult people with eating disorders. Psychoneuroendocrinology, 115(December 2019), 104644. 10.1016/j.psyneuen.2020.104644 32171902

[erv2896-bib-0030] Monteleone, A. M. , Monteleone, P. , Serino, I. , Scognamiglio, P. , Di Genio, M. , & Maj, M. (2015). Childhood trauma and cortisol awakening response in symptomatic patients with anorexia nervosa and bulimia nervosa. International Journal of Eating Disorders, 48(6), 615–621. 10.1002/eat.22375 25808182

[erv2896-bib-0031] Monteleone, A. M. , Monteleone, P. , Volpe, U. , De Riso, F. , Fico, G. , Giugliano, R. , Nigro, M. , & Maj, M. (2018). Impaired cortisol awakening response in eating disorder women with childhood trauma exposure: Evidence for a dose‐dependent effect of the traumatic load. Psychological Medicine, 48(6), 952–960. 10.1017/S0033291717002409 28847330

[erv2896-bib-0032] Pan, X. , Kaminga, A. C. , Wen, S. W. , Wang, Z. , Wu, X. , & Liu, A. (2020). The 24‐hour urinary cortisol in post‐traumatic stress disorder: A meta‐analysis. PLoS One, 15(1), 1–15. 10.1371/journal.pone.0227560 PMC6952249 31918435

[erv2896-bib-0033] Rienecke, R. D. , Blalock, D. V. , Duffy, A. , Manwaring, J. , Le Grange, D. , Johnson, C. , Mehler, P. S. , & McClanahan, S. F. (2021). Posttraumatic stress disorder symptoms and trauma‐informed care in higher levels of care for eating disorders. International Journal of Eating Disorders, 54(4), 627–632. 10.1002/eat.23455 33382109

[erv2896-bib-0034] Schmidt, U. , Humfress, H. , & Treasure, J. (1997). The role of general family enviroment and sexual and physical abuse in the origins of eating disorders. European Eating Disorders Review, 5(3), 184–207.

[erv2896-bib-0035] Schnurr, P. , Vielhauer, M. , Weathers, F. , & Findler, M. (1999). The brief trauma questionnaire (BTQ) [measurement instrument]. National Center for PTSD. Retrieved from https://www.ptsd.va.gov/professional/assessment/documents/BTQ.pdf

[erv2896-bib-0036] Schumacher, S. , Niemeyer, H. , Engel, S. , Cwik, J. C. , Laufer, S. , Klusmann, H. , & Knaevelsrud, C. (2019). HPA axis regulation in posttraumatic stress disorder: A meta‐analysis focusing on potential moderators. Neuroscience & Biobehavioral Reviews, 100(June 2018), 35–57. 10.1016/j.neubiorev.2019.02.005 30790632

[erv2896-bib-0037] Teicher, M. H. , & Samson, J. A. (2013). Childhood maltreatment and psychopathology: A case for ecophenotypic variants as clinically and neurobiologically distinct subtypes. American Journal of Psychiatry, 170(10), 1114–1133. 10.1176/appi.ajp.2013.12070957 23982148 PMC3928064

[erv2896-bib-0038] Todisco, P. , Meneguzzo, P. , Garolla, A. , Antoniades, A. , Vogazianos, P. , & Tozzi, F. (2020). Impulsive behaviors and clinical outcomes following a flexible intensive inpatient treatment for eating disorders: Findings from an observational study. Eating and Weight Disorders, 26(3), 869–877, 0123456789. 10.1007/s40519-020-00916-5 32430886

[erv2896-bib-0039] Walker, E. A. , Unutzer, J. , Rutter, C. , Gelfand, A. , Saunders, K. , VonKorff, M. , Bernstein, B. , & Katon, W. (1999). Costs of health care use by women HMO members with a history of childhood abuse and neglect. Archives of General Psychiatry, 56(7), 609–613. 10.1001/archpsyc.56.7.609 10401506

